# Body composition changes and clinical outcomes in pediatric cystic fibrosis during 24 months of lumacaftor ivacaftor therapy based on real-world data

**DOI:** 10.1038/s41598-025-86010-1

**Published:** 2025-01-17

**Authors:** Marcell Imrei, Adrienn F. Kéri, Éva Gács, Ildikó Gönczi, Melinda Meláth, Éva Kosaras, Botond Demeter, Csaba Péterfia, Klára Vass, Gyöngyi Székely, Klementina Ocskay, Andrea Párniczky

**Affiliations:** 1 Heim Pál National Pediatric Institute, Budapest, Hungary; 2 Institute for Translational Medicine, Medical School, University of Pécs, Pécs, Hungary; 3 Velkey László Child’s Health Center, Borsod-Abaúj-Zemplén County Central Hospital and University Teaching Hospital, Miskolc, Hungary; 4 Department of Pediatrics, Medical School, University of Pécs, Pécs, Hungary; 5 Jósa András Hospital, Szabolcs-Szatmár-Bereg County Hospitals and University Teaching Hospital, Nyíregyháza, Hungary; 6 Pediatric Rehabilitation Unit, Kaposi Mór Teaching Hospital, Mosdós, Hungary; 7 Pharmaceutical Sciences and Health Technologies Division, Doctoral School, Semmelweis University, Budapest, Hungary

**Keywords:** Normal-weight obesity, Fecal elastase, Pancreatic function, Body composition, CFTR modulator therapy, LUM/IVA, Paediatric research, Clinical trials, Cystic fibrosis

## Abstract

Clinical trials demonstrate the short-term efficacy of dual CFTR modulators, but long-term real-world data is limited. We aimed to investigate the effects of 24-month lumacaftor/ivacaftor (LUM/IVA) therapy in pediatric CF patients (pwCF). This observational study included pwCF homozygous for F508del mutation treated between 2021 and 2023. We report data for the first 24 months from therapy initiation. Variables were analyzed separately for ages 2–5, 6–11, and over 12. Data from 49 pwCF (median age: 9.3 years (5.5–14.2)) showed that ppFEV1 values after a transient increase at 12 months, decreased from 102% (82–114) at baseline to 87% (74–96) at 24 months. The decrease was more pronounced with higher initial ppFEV1. Median sweat chloride concentration decreased from 75 mmol/L (69–82) to 57 mmol/L (43–70) without any association with respiratory function change. Median BMI *z*-score increased from − 0.81 (− 1.37–0.49) to − 0.39 (− 0.88 to  − 0.04) (*p* = 0.288), and the proportion of underweight and overweight children decreased. Skeletal muscle mass remained stable, while fat mass significantly increased (*p* = 0.011). Fecal elastase levels improved, especially among younger patients. These findings underscore the potential benefits of early initiation of CFTR modulator therapy in pediatric CF patients, highlighting improvements in nutritional status and pancreatic function.

## Introduction

Over the past decade, the emergence of cystic fibrosis transmembrane conductance regulator (CFTR) modulator therapies significantly transformed cystic fibrosis (CF) care, and they have become a key area of research^1^. The lumacaftor/ivacaftor (LUM/IVA) combination (marketed as Orkambi®) is approved for patients with cystic fibrosis (pwCF) homozygous for F508del from the age of one year onwards. The efficacy and safety of this therapy have been confirmed in several phase 3 trials^2–8^, leading to further investigations focused on specific patient groups, long-term outcomes, rare side effects, and extrapulmonary outcomes (e.g. weight gain, glucose tolerance, microbiome).

Pre-modulator studies have shown, that higher body mass index (BMI) and fat-free mass correlate with the percentage predicted forced expiratory volume in the first second (ppFEV1)^9,10^, but normal-weight obesity in the CF population is associated with lower ppFEV1 compared to obese and overweight pwCF^9^. Although several studies explored body composition changes after the initiation of CFTR modulator therapy in adults^11–13^, peer-reviewed pediatric follow-up data assessing the effects of modulator therapy on body composition, particularly fat mass and its correlation with clinical outcomes are lacking. Short follow-up times limit our understanding in regard to the long-term real-world effects of modulator usage.

In light of these knowledge gaps, we aimed to investigate the effects of 24 months of LUM/IVA therapy focusing on changes in body composition besides ppFEV1 and sweat chloride concentration in children with CF.

## Methods

### Study design

Our real-world study analyzes data derived from a subset of Heim Pál National Pediatric Institute’s comprehensive data collection on the general pediatric CF population of the center. In this study, we examined data exclusively from the patients who received LUM/IVA therapy.

The data collection was approved by the Scientific and Research Ethics Committee of the Hungarian Medical Research Council (23508-5/2018/EÜIG). Informed consent has been obtained from the parent and/or legal guardian for study participation. All patients provided written informed consent. The study was conducted following the ethical principles of the World Medical Association Declaration of Helsinki^14^.

### Population

We included all patients who were diagnosed with cystic fibrosis (CF) in accordance with the current guidelines^15^, were homozygous for the F508del mutation, were at least 2 years old (the minimum age for initiating LUM/IVA therapy at that time), and began LUM/IVA therapy between February and October 2021 in the analysis.

We conducted subgroup analysis based on age, dividing participants into three groups: 2–5 years, 6–11 years, and 12 years or older, following the age limits for LUM/IVA dosages as stated in the European Medicines Agency’s Summary of Product Characteristics^16^. Patients are referred to adult care between the ages of 18 and 21, leading the clinic to initiate CFTR modulator therapy up to age 21.

### Examination and outcomes

The therapy was initiated during a 5–7 days long hospitalization, followed by outpatient visits scheduled at 1 week, 1 month, and 3 months post-treatment initiation, followed by subsequent appointments every 3 months. In instances of laboratory abnormalities or notable side effects, the multidisciplinary team deliberated on dosage modification or discontinuation. The tests outlined in Supplementary Table 1 were performed during outpatient visits.

### Outcomes

The percentage predicted forced vital capacity (ppFVC) and ppFEV1 were evaluated—commencing between the ages of 5 and 8 years, based on the child’s capability. Lung function results from the three years prior to the initiation of CFTR modulator treatment were used for comparison.

Sweat samples were collected with the Macroduct® Advanced kit and chloride concentration was measured after pilocarpine iontophoresis using the Chlorochek® chloridometer (both manufactured by EliTech Group Inc., Paris, France).

We investigated changes in height, weight, and BMI *z*-score values standardized for age and sex. Participants were categorized as obese, overweight, normal-weight, or underweight according to the ESPEN-ESPGHAN-ECFS guideline on nutrition care for cystic fibrosis^17^.

Body composition parameters (total body water (TBW), fat mass (FM), skeletal muscle mass (SMM), body protein content (BPC), and body mineral content (BMC)) were measured via bioelectrical impedance analysis (BIA) with InBody770 (InBodyUSA, Cerritos, CA), a reliable tool for the assessment of body composition changes^18^.

To monitor hepatic status before and during LUM/IVA therapy, aspartate transaminase (AST), alanine transaminase (ALT), γ-glutamyl transferase (GGT), alkaline phosphatase (ALP), and direct bilirubin levels were measured at fixed intervals before and after the start of therapy.

Fecal elastase was measured every 6 months. Patients were classified as pancreas exocrine insufficient (PEI) with fecal elastase level measured 200 µg/g or less, and pancreas exocrine sufficient (PES) if levels exceeded 200 µg/g. PEI severity was mild for elastase levels between 100 and 200 µg/g, and severe if below 100 µg/g^19^.

At each visit, either a sputum culture was performed, or, if the patient could not produce sputum, a throat swab was taken. Pre-treatment sputum microbiological findings were also analyzed to assess changes during modulator therapy. To quantify the extent of *Pseudomonas aeruginosa* colonization, all patients were categorized according to the Leeds criteria^20^ for the one-year period prior to therapy and the second year of therapy.

### Statistical analysis

For height, weight, and BMI values we used *z*-scores. Sex- and age-standardized *z*-scores were calculated based on LMS parameters using the 2000 Centers for Disease Control and Prevention reference^21^. TBW, FM, SMM, BPC, BMC are reported as percentage of weight.

We utilized a paired *t*-test to examine the changes in various continuous variables across follow-up visits, while the Kruskal–Wallis rank sum test was employed to compare the results of these continuous variables among different subgroups. Fisher’s exact test was employed to compare categorical variables among different follow-up visits and age groups. The initial ppFEV1 and its variation and the initial sweat chloride concentration and its variation were compared by Q-Q plots, and the optimal fit was determined by a linear function or, in case of clear skewness of the data, by a second-order polynomial function via least squared approximation. Values are expressed in median and interquartile range. The result was considered significant if the *p*-value was less than 0.05. All analyses were carried out by R version 4.1.0.

## Results

At the time of data collection, our center provided care for 54 children eligible for LUM/IVA therapy, of whom 49 started the therapy. At the end of the 2-year follow-up, 42 children remained in the analyzed cohort (for a detailed flow diagram see Supplementary Fig. 1).

The baseline characteristics of the three age subgroups are shown in Table 1. Significant differences in ppFEV1, ppFVC, and CFRD are age-specific. Anthropometric parameters, lung function, sweat chloride concentration, and body composition parameters at baseline and 24 months are shown in Table 2. Data completeness for Tables 1 and 2 are shown in Supplementary Table 3.

**Table 1:** Baseline characteristics.

	All patients	2–5 years old	6–11 years old	≥ 12 years old	*p*-value
n	49	15	14	20	
Number of boys (%)	57	47	50	70	0.315
Age at diagnosis (months)*	8.9 (3.4, 24.2)	11.5 (3.6, 22.0)	6.4 (1.8, 20.1)	10.0 (4.7, 24.9)	0.367
Age at the start of LUM/IVA (years)*	9.3 (5.5, 14.2)	4.6 (3.5, 5.2)	8.5 (7.9, 9.5)	16.2 (13.5, 17.2)	
Sweat chloride (mmol/L)*	74 (67, 86)	70 (66, 78)	73 (62, 77)	83 (69, 92)	0.154
ppFEV1 (%)*	94 (79, 112)	–	109 (103, 122)	82 (70, 96)	**0.001**
ppFVC (%)*	103 (87, 118)	–	112 (98, 122)	93 (78, 110)	**0.037**
Fecal elastase (µg/g)					1.000
< 100	39 (98%)	13 (100%)	12 (100%)	14 (93%)	
100–200	0 (0%)	0 (0%)	0 (0%)	0 (0%)	
> 200	1 (2%)	0 (0%)	0 (0%)	1 (7%)	
CFRD	4/49 (8%)	0/15 (0%)	0/14 (0%)	4/20 (20%)	**0.034**
P. aeruginosa colonization					0.276
Never	10 (21%)	3 (20%)	3 (21%)	4 (21%)	
Free	19 (40%)	4 (27%)	6 (43%)	9 (47%)	
Intermittent	14 (29%)	8 (53%)	3 (21%)	3 (16%)	
Chronic	5 (10%)	0 (0%)	2 (14%)	3 (16%)	

*LUM/IVA* lumacaftor/ivacaftor, *ppFEV1* percentile predicted forced expiratory volume in the first second, *ppFVC* percentile predicted forced vital capacity, *CFRD* cystic fibrosis-related diabetes.

*median, interquartile range.

Significant values are in bold.

**Table 2:** Anthropometric data, lung function, sweat chloride concentration, and body composition parameters at baseline and 24 months of LUM/IVA treatment of patients who completed the follow-up period.

Characteristic	At baseline				24 months of LUM/IVA				*p*-value
	Overall	2–5 years old	6–11 years old	≥ 12 years old	Overall	2–5 years old	6–11 years old	≥ 12 years old	
Weight z-score*	− 0.54 (− 1.11, 0.16)	− 0.25 (− 1.03, 0.65)	− 0.32 (− 0.76, 0.14)	− 1.06 (− 1.86, − 0.11)	− 0.19 (− 0.84, 0.19)	− 0.19 (− 0.47, 0.54)	− 0.07 (− 0.79, 0.21)	− 0.50 (− 1.50, − 0.01)	0.074
Height z-score*	− 0.31 (− 1.04, 0.62)	− 0.06 (− 0.69, 0.60)	0.20 (− 0.65, 0.82)	− 0.65 (− 1.38, 0.01)	− 0.13 (− 0.68, 0.65)	− 0.20 (− 0.67, 0.23)	0.08 (− 0.69, 1.00)	− 0.13 (− 0.55, 0.18)	**0.042**
BMI z-score*	− 0.81 (− 1.37, 0.49)	− 0.29 (− 1.44, 0.79)	− 0.45 (− 0.93, 0.69)	− 1.00 (− 1.65, − 0.71)	− 0.39 (− 0.88, − 0.04)	− 0.03 (− 0.51, 0.65)	− 0.40 (− 0.82, − 0.12)	− 0.61 (− 1.53, − 0.23)	0.288
ppFEV1 (%)*	102 (82, 114)	–	112 (104, 122)	83 (72, 98)	87 (74, 96)	–	96 (88, 103)	76 (68, 88)	** < 0.001**
Sweat chloride (mmol/L) *	75 (69, 82)	69 (64, 78)	75 (70, 77)	79 (68, 90)	57 (43, 70)	49 (37, 63)	60 (48, 76)	57 (44, 68)	** < 0.001**
Fecal elastase (µg/g)									0.085
< 100	28 (97%)	9 (100%)	10 (100%)	9 (90%)	23 (79%)	6 (67%)	9 (90%)	8 (80%)	
100–200	0 (0%)	0 (0%)	0 (0%)	0 (0%)	2 (7%)	1 (11%)	0 (0%)	1 (10%)	
> 200	1 (3%)	0 (0%)	0 (0%)	1 (10%)	4 (14%)	2 (22%)	1 (10%)	1 (10%)	
Body composition (%)*									
Total body water	63 (59, 66)	63 (62, 66)	62 (58, 65)	64 (61, 67)	62 (57, 65)	60 (55, 63)	59 (58, 62)	64 (62, 68)	0.051
Body protein content	17 (16, 18)	17 (16, 17)	16 (16, 18)	17 (16, 18)	17 (15, 17)	16 (14, 17)	16 (15, 17)	17 (17, 19)	0.087
Fat mass	11 (9, 17)	8 (3, 17)	15 (10, 17)	11 (9, 16)	15 (12, 22)	19 (15, 25)	18 (15, 21)	12 (8, 14)	**0.012**
Skeletal muscle mass	43 (39, 47)	40 (38, 43)	43 (39, 45)	48 (42, 50)	42 (40, 49)	39 (34, 42)	41 (40, 45)	49 (46, 51)	0.714

*LUM/IVA* lumacaftor/ivacaftor, *BMI* body mass index, *ppFEV1* percentile predicted forced expiratory volume in the first second.

*median, interquartile range.

Significant values are in bold.

### Lung function

At the initiation of LUM/IVA therapy, ppFEV1 and ppFVC of almost half of the patients exceeded 100% of the predicted value. In the 6–11 years age group, the median ppFEV1 was 109% (IQR: 103–122), indicating most of the values were above the normal reference. Conversely, children aged 12 years or older had a median ppFEV1 of 82% (IQR: 70–96), suggesting worse lung function than the average of the reference population (Table 1).

At 24 months, the lung function (ppFEV1) of the 6–11 age group decreased and was no longer different from the reference population (median: 112, IQR: 104–122 at baseline and median: 96, IQR: 88–103 at 24 months). A more modest respiratory function decline was observed in the ≥ 12-year-old population (median: 83, IQR: 72–98 at baseline and median: 76, IQR: 68–88 at 24 months) (Table 2).

**Fig. 1:**
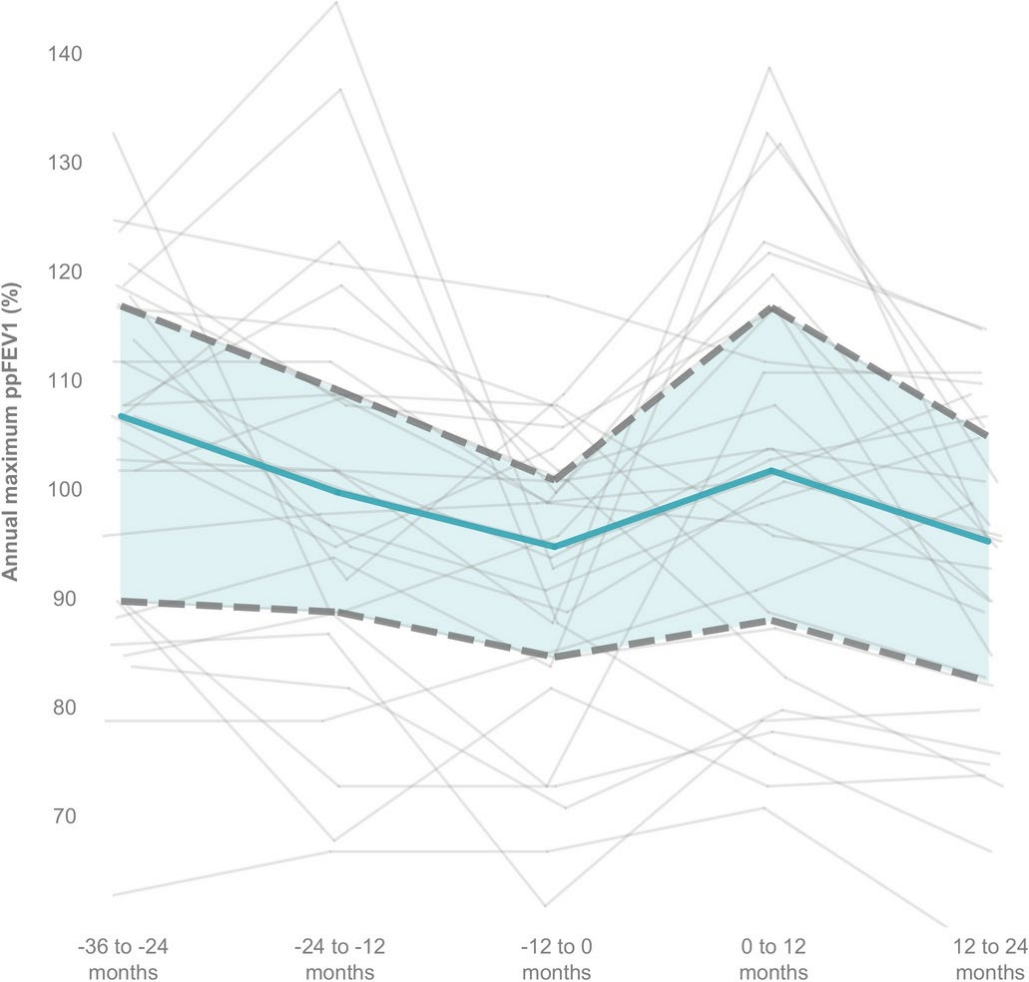
The highest annual ppFEV1 values in the three years prior to therapy initiation and during the first and second years of LUM/IVA therapy. Solid blue line represents the median value, dashed lines represent the 1st and 3rd quartiles *(ppFEV1: percentile predicted forced expiratory volume in the first second).*

Examining the highest annual ppFEV1 values in the three years prior to therapy and during therapy, a transient increase was seen in the first year of therapy (Fig. 1).

**Fig. 2:**
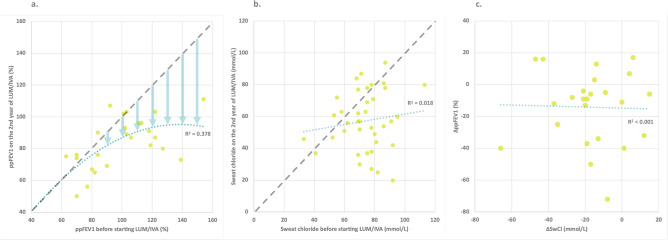
(**a**) Association between the initial ppFEV1 and the ppFEV1 after 24 months of LUM/IVA therapy (**b**) Association between the initial sweat chloride concentration and levels after 24 months of LUM/IVA therapy (**c**) Association between the change of ppFEV1 and the change of sweat chloride concentration during the 24-month LUM/IVA therapy. Dotted lines represent the trend lines, arrows represent the greater reduction at higher initial values. (LUM/IVA: lumacaftor/ivacaftor; ppFEV1: percentile predicted forced expiratory volume in the first second).

We observed a noteworthy trend in the initial ppFEV1 and its subsequent change. We found that individuals with higher initial ppFEV1 experienced a proportionally greater reduction in ppFEV1 over the course of two years (*r*^2^ = 0.378) (see Fig. 2a).

### Changes in sweat chloride concentrations

Median sweat chloride concentrations decreased from 75 mmol/L at baseline to 57 mmol/L at 24 months (Table 2). However, Europe-wide shortage of Macroduct® sweat collection kits resulted in missing data (17%, Supplementary Table 3). Interestingly, no correlation was found between initial sweat chloride concentrations and those measured at 24 months (*r*^2^ = 0.018) (Fig. 2b). 24-month sweat chloride concentrations scattered around 50–60 mmol/L regardless of the level measured before therapy (Fig. 2b), falling in the intermediate range, well above the cut-off of 29 mmol/L for healthy individuals.

The change in ppFEV1 and the change in sweat chloride concentration were not correlated (*r*^2^ < 0.001) (Fig. 2c).

### Anthropometry and body composition

At the initiation of LUM/IVA therapy, the age- and sex-adjusted weight, height, and BMI *z*-scores were lower in all age groups compared to the reference population, and pwCF ≥ 12-year-old had the lowest z-scores (Table 2).

**Fig. 3:**
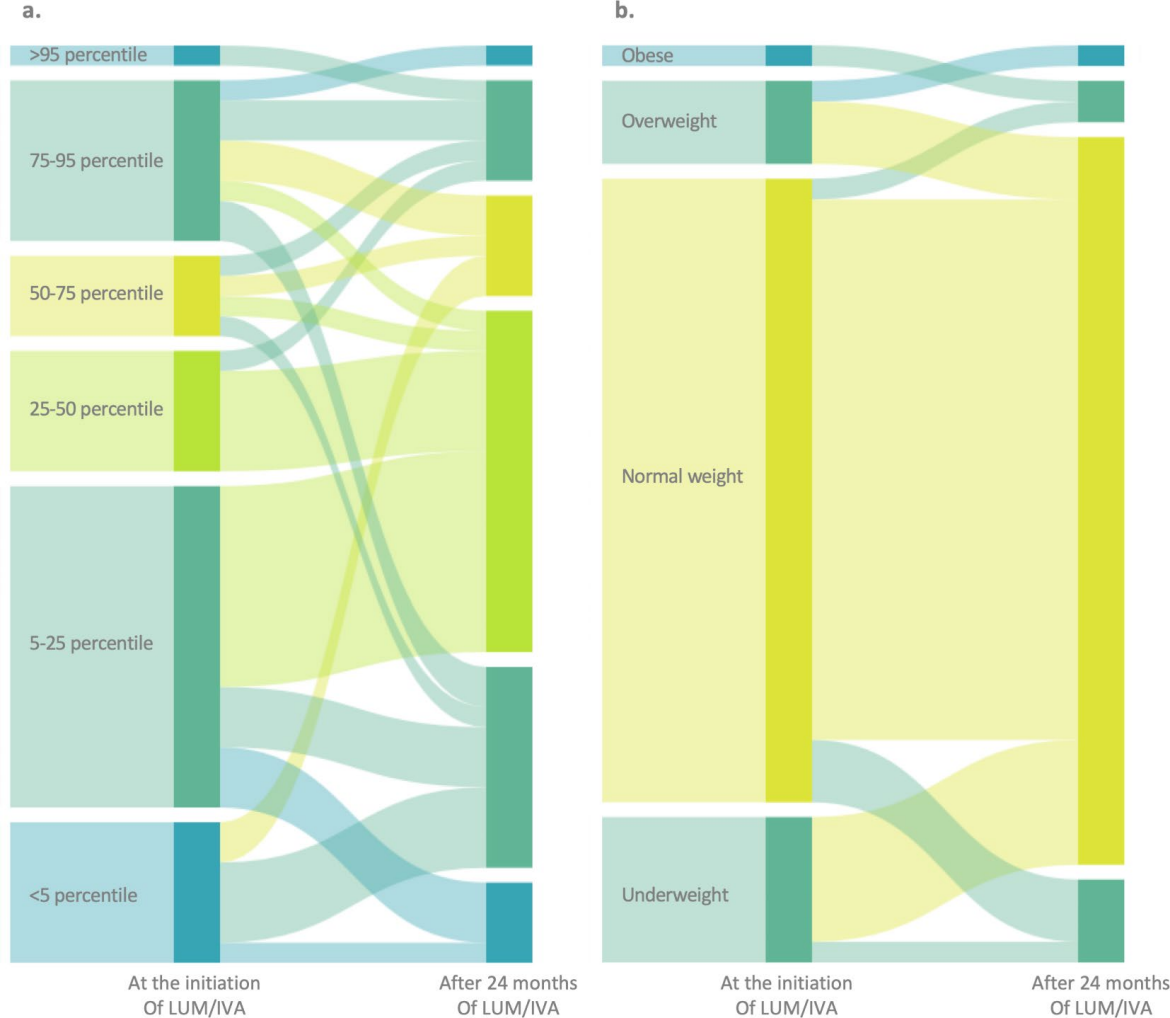
Distribution of participants by (**a**) BMI percentile and (**b**) BMI categories at the initiation of LUM/IVA therapy and at 24 months (LUM/IVA: lumacaftor/ivacaftor).

Over the 24 months, a noteworthy shift was observed in BMI percentiles among the children. There was an evident increase in the number of children in the 25–75 percentile range, while there was a decline in those below the 25th percentile or above the 75th percentile (Fig. 3a). Initially, 7 children were classified as underweight, 30 as normal weight, four as overweight, and one as obese based on the current nutritional care guidelines for CF (Fig. 3b). At 24 months, the number of underweight children decreased to four, while the number of normal-weight children increased to 35. The count of overweight children was two, and one child remained classified as obese. Despite the improvements in BMI, weight, and height *z*-scores over the two-year period, more than half of the participants still had measurements below the mean of the reference population by the study’s end, as delineated in Table 2.

No significant changes were observed in TBW%, BPC%, and SMM% during the 2 years of the study. We observed a substantial rise of the FM% from baseline to 24 months (*p* = 0.012, Table 2), which was particularly remarkable in the 2-to-5-year-old population, where FM% increased more than twofold (8% (IQR: 3–17%) at baseline; 19% (IQR: 15–25%) at 24 months) over the 2 years without the increase of any other body composition parameters.

### Laboratory parameters

Transaminase levels remained in the normal range except for one patient. A temporary (3-month) suspension of LUM/IVA therapy was required in this case at the 3^rd^ month of therapy.

Bilirubin levels were below the detection limit at baseline and during the analyzed period in almost all patients and never exceeded the normal range, while ALP showed significant decreases (Supplementary Table 2–3).

Only one patient was PES at baseline, while at 24 months four children were PES and two others had fecal elastase levels between 100 and 200 ug/g. The greatest improvement was seen in the 2-to-5-year age group (Supplementary Fig. 2–3).

### Pseudomonas aeruginosa colonization

At baseline, according to the Leeds criteria, 5 pwCF were chronically colonized and 13 pwCF had intermittent *Pseudomonas aeruginosa* colonization. At 24 months, 5 participants’ status was chronic colonization and 10 intermittent (*p* = 0.655). For detailed data before and after therapy according to Leeds criteria, see Supplementary Fig. 4.

## Discussion

This single-center real-world observational study examined the effects of 24-month LUM/IVA therapy on pediatric patients with CF, comprising 49 individuals. This cohort represents approximately 45% of the total eligible pediatric CF population in Hungary, based on data reported in the Hungarian Cystic Fibrosis Registry^22^ and the most recent annual report of the ECFS Patient Registry^23^.

Prior to therapy initiation, a considerable portion of our patients exhibited ppFEV1 values exceeding 100%, with the majority also surpassing this threshold for ppFVC. This indicates a remarkably higher baseline respiratory function compared to previous LUM/IVA studies^2,7,24^. Transient spikes in annual maximum ppFEV1 values were noted in the first year, after which a decline in annual maximum ppFEV1 was observed. This transient improvement may reflect the initial benefits of improved CFTR function and mucus clearance facilitated by LUM/IVA therapy; however, the subsequent decline suggests that the therapy may not adequately suppress the persistent inflammatory and fibrotic remodeling processes^25^ in the lungs over the long term. These findings align with observations that highly effective modulator therapies, such as elexacaftor/tezacaftor/ivacaftor, provide superior stabilization of lung function and broader clinical benefits^26,27^.

We observed a positive correlation between the rate of ppFEV1 reduction by the second year and pre-treatment ppFEV1 levels. This correlation likely contributes to the average annual decline in ppFEV1 observed in our cohort, reaching 7.4%, which exceeds the reductions reported in prior studies^2,7,24^ with lower initial ppFEV1 values (60–90%).

The baseline sweat chloride concentrations of our patients were lower than the levels typically observed in phase 3 trials ranging from 100 to 110 mmol/L^5,6^. Consequently, these patients exhibited a smaller decline in sweat chloride concentrations over the 2-year follow-up period. This pattern aligns with our observation that patients with lower baseline values tended to experience less pronounced declines, as 24-month sweat chloride concentration was not correlated with the initial value. This finding is consistent with previous observations made over shorter follow-up periods^24,28,29^.

We did not observe an association between the reduction in sweat chloride concentrations and the change in ppFEV1, similar to studies reported on 6 months LUM/IVA therapy^30,31^.

In line with several previous studies^2,24,32,33^, standardized anthropometric data (height, weight, and BMI) were slightly lower at baseline compared to the healthy population and then showed a substantial increase during follow-up. Although overall BMI *z*-score did not increase significantly, BMI seemed to normalize across patients: those with initial low BMI showed an increase in their *z*-scores, while patients with high BMI experienced a decrease (Fig. 3). This observation is similar to, but more pronounced than, those reported in some previous studies^24,34^. As reported by Dress et al. and Kim et al., children on CFTR modulators have higher BMI z-scores compared to those without therapy^35,36^, but BMI is a very unspecific indicator and provides relatively little information on nutritional status^11^. Notably, respiratory function shows a strong correlation with fat-free mass, while it is not associated^37^ or even negatively correlated with fat mass^9^. Based on this information, although high BMI correlates with better respiratory function, body composition seems to more adequately describe patients’ condition^10,38^. Changes in body composition during CFTR modulator therapy have so far only been studied in adults, furthermore, only one study has compared children with and without modulator therapy^35^. In adult studies, there was a near-unanimous observation that fat mass increased with LUM/IVA and IVA therapy, whereas fat-free mass remained largely unchanged^12,13^.

We found that the relative increase in body weight is not driven by a proportional accumulation of fat and muscle, but almost entirely by fat gain. This phenomenon is particularly noticeable in the youngest age group, where the proportion of body fat increased more than twofold over 2 years, while the proportion of skeletal muscle mass remained unchanged.

An increase in fecal elastase was observed during therapy. By the end of the follow-up period, more than 10% of children had an elastase value above 200 mg/g. Pancreatic enzyme replacement therapy dosage could be reduced in one case during the observation period. This observation is similar to that seen in the phase 3 studies of LUM/IVA involving children under 5 years^3–5^ and subsequent studies^39^. In line with these previous observations^39,40^, our data also indicate that modulator therapy started at a younger age may lead to better results in restoring pancreatic exocrine function.

Interestingly, liver function tests showed a statistically significant decrease, consistent with previous studies and potentially indicating additional extrapulmonary benefits of LUM/IVA therapy^41,42^.There are several strengths to this study. Firstly, we achieved almost complete inclusion of the target population, encompassing 91% of eligible children for therapy, representing a significant portion (45%) of the Hungarian pediatric population eligible for such treatment. Notably, our study participants exhibited particularly favorable baseline characteristics compared to similar investigations, which provides valuable insights into the influence of initial clinical status on the anticipated outcomes of CFTR modulator therapy. Additionally, our study stands out due to its extensive follow-up period of 24 months. We also expanded upon previous research by examining several parameters previously unexplored in larger studies, including body composition parameters and Leeds criteria. The comprehensive nature of our data collection is noteworthy, and the analysis benefitted from minimal missing data, enhancing the reliability of our findings.

However, certain limitations should be acknowledged. Technical constraints restricted the measurement of parameters such as respiratory function and body composition to older children, potentially introducing bias and limiting the generalizability of our findings. Additionally, the decrease in sputum production, and consequently, the reduction in lower airway samples during CFTR modulator therapy posed challenges in evaluating microbiological results. This limitation may have biased the interpretation of microbiological data and warrants caution when interpreting these findings. Moreover, the cohort’s lower sweat chloride concentrations and higher baseline ppFEV1 reflect a milder disease severity compared to broader CF populations, which may influence the observed therapeutic effects and reduce comparability to other studies.

In summary, 24-month LUM/IVA therapy resulted in transiently increased, then declined lung function, significant reductions in sweat chloride concentrations, and substantial increases in BMI percentile, primarily driven by fat gain. Additionally, fecal elastase levels improved, particularly in younger patients. These findings underscore the potential benefits of early initiation of CFTR modulator therapy in pediatric CF patients, particularly in improving nutritional status and pancreatic function.

## Supplementary Information

Supplementary Information.

## Data Availability

Data that support the findings of the study are available on request from the corresponding author (A.P.). The data are not publicly available due to ethical restrictions.
