# Supplementary material for: Body composition changes and clinical outcomes in pediatric cystic fibrosis during 24 months of lumacaftor ivacaftor therapy based on real-world data
Source: Sci Rep. 2025 Jan 17;15:2247. doi: 10.1038/s41598-025-86010-1 (PMC11748628; doi:10.1038/s41598-025-86010-1)

Shaping a Healthier Future: Body Composition Changes and Clinical Improvements in Pediatric Cystic Fibrosis Patients on Lumacaftor/Ivacaftor Therapy: a 24-month Real-World Study

Marcell Imrei[1,2] (marcell.imrei@gmail.com), Adrienn F. Kéri[1] (adrikeri@gmail.com), Éva Gács[1] (evagacs@hotmail.com), Ildikó Gönczi[1] (gonczi.ildiko@heimpalkorhaz.hu), Melinda Meláth[1] (melinda.melath@heimpalkorhaz.hu), Éva Kosaras[3] (kosaras.vica@gmail.com), Botond Demeter[3] (demeter.botond@gmail.com) , Csaba Péterfia[4] (peterfia.csaba@pte.hu), Klára Vass[5] (vass.klara@t-online.hu), Gyöngyi Székely[6] (sz.gyongyi58@gmail.com), Klementina Ocskay[1,7] (ocskay.klementina@gmail.com), Andrea Párniczky[1,2,7] * (andrea.parniczky@gmail.com)

Suppl. Table 1 Study assessment schedule *table (GOT: glutamic oxaloacetic transaminase, GPT: glutamic-pyruvic transaminase, GGT: gamma-glutamyltransferase, ALP: alkaline phosphatase)*

	Before initiation	1 week	1 month	3 month	6 month	9 month	12 month	15 month	18 month	21 month	24 month
Weight	x		x	x	x	x	x	x	x	x	x
Height	x		x	x	x	x	x	x	x	x	x
Lung function	x	x	x	x	x	x	x	x	x	x	x
Body composition	x				x		x		x		x
Fecal elastase (µg/g)	x				x		x		x		x
Sweat chloride concentration (mmol/L)	x				x		x		x		x
GOT (U/L)	x	x	x	x	x	x	x		x		x
GPT (U/L)	x	x	x	x	x	x	x		x		x
GGT (U/L)	x	x	x	x	x	x	x		x		x
ALP (U/L)	x	x	x	x	x	x	x		x		x
Airway culture	x	x	x	x	x	x	x	x	x	x	x

Suppl. Table 2 Results of the serum markers at the initiation of the therapy and after the 2-year follow-up *(GOT: glutamic oxaloacetic transaminase, GPT: glutamic-pyruvic transaminase, GGT: gamma-glutamyltransferase, ALP: alkaline phosphatase)*

	**Initiation**				**After 2 years**				**p**
	overall	2-5 year old	6-11 year old	>12 year old	overall	2-5 year old	6-11 year old	>12 year old	
**GOT (U/L)**	30 (23-37)	34 (32.5-39.5)	29 (27-39.5)	23 (20-26.5)	30 (23-34)	36 (30-39)	27 (23.5-30)	24 (21-31.5)	0.1622
**GPT (U/L)**	25.5 (20.25-32)	26 (23.25-30.25)	34 (22.75-44.25)	21.5 (18.25-28)	21.5 (16.25-28)	27 (15.5-33.5)	23.5 (19.75-26.5)	19.5 (15.25-24)	0.1038
**GGT (U/L)**	12 (10-19)	10 (9.25-11)	14.5 (11-30.5)	13.5 (10-20.25)	10.5 (10-15)	10 (10-12.5)	12.5 (10-16)	10.5 (8.75-15.25)	**0.0395**
**ALP (U/L)**	282 (208-354)	277 (233-332)	364 (264-422)	239 (126-295)	231 (125-274)	250 (193-275)	268 (225-307)	141 (75-208)	**<0.0001**

Suppl. Table 3 Data completeness *(ppFEV1: percentile predicted forced vital capacity of the first second, ppFVC: percentile predicted forced vital capacity, PEI: pancreas exocrine insufficiency, CFRD: cystic fibrosis-related diabetes, BMI: body mass index, GOT: glutamic oxaloacetic transaminase, GPT: glutamic-pyruvic transaminase, GGT: gamma-glutamyltransferase, ALP: alkaline phosphatase)*

**Data completeness for Table 1**		
Sweat chloride concentration	49/49	100%
ppFEV1 (%)	32/40	80%
ppFVC (%)	32/40	80%
PEI	49/49	100%
CFRD	49/49	100%
P. aeruginosa colonization	46/49	94%
**Data completeness for Table 2**		
Weight z-score	42/42	100%
Height z-score	42/42	100%
BMI z-score	42/42	100%
ppFEV1 (%)	24/33	73%
Sweat chloride (mmol/L)	35/42	83%
Fecal elastase (µg/g)	29/42	69%
Body composition	30/42	71%
**Data completeness for Supplementary Table 2**		
GOT (U/L)	37/42	88%
GPT (U/L)	42/42	100%
GGT (U/L)	42/42	100%
ALP (U/L)	40/42	95%

Suppl. Figure 1 Study flowchart

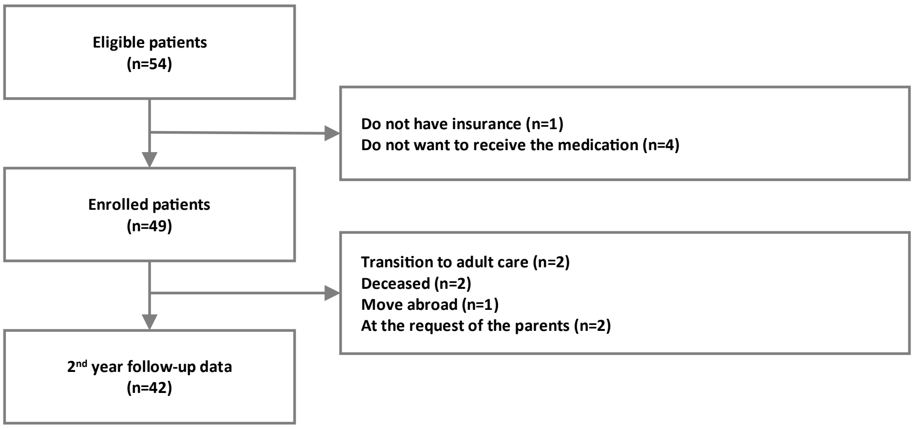

Suppl. Figure 2 Pancreas exocrine status at the initiation of the therapy and after 24 months of LUM/IVA therapy

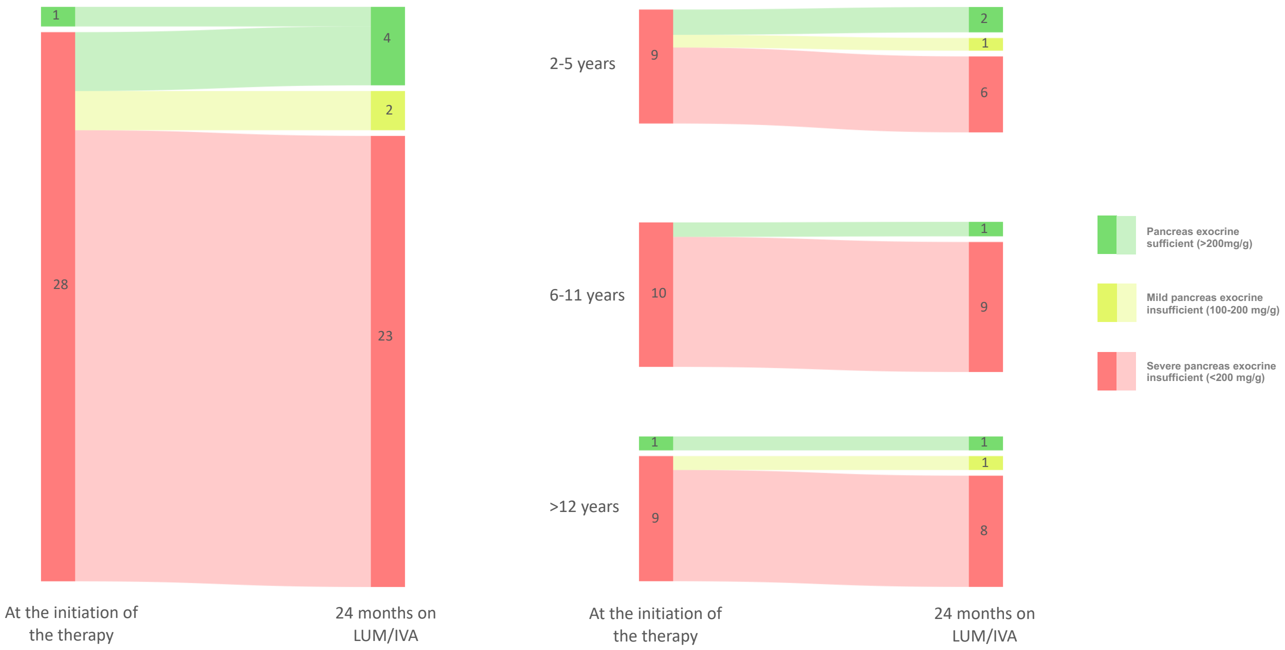

Suppl. Figure 3 Change of fecal elastase level during the follow-up period

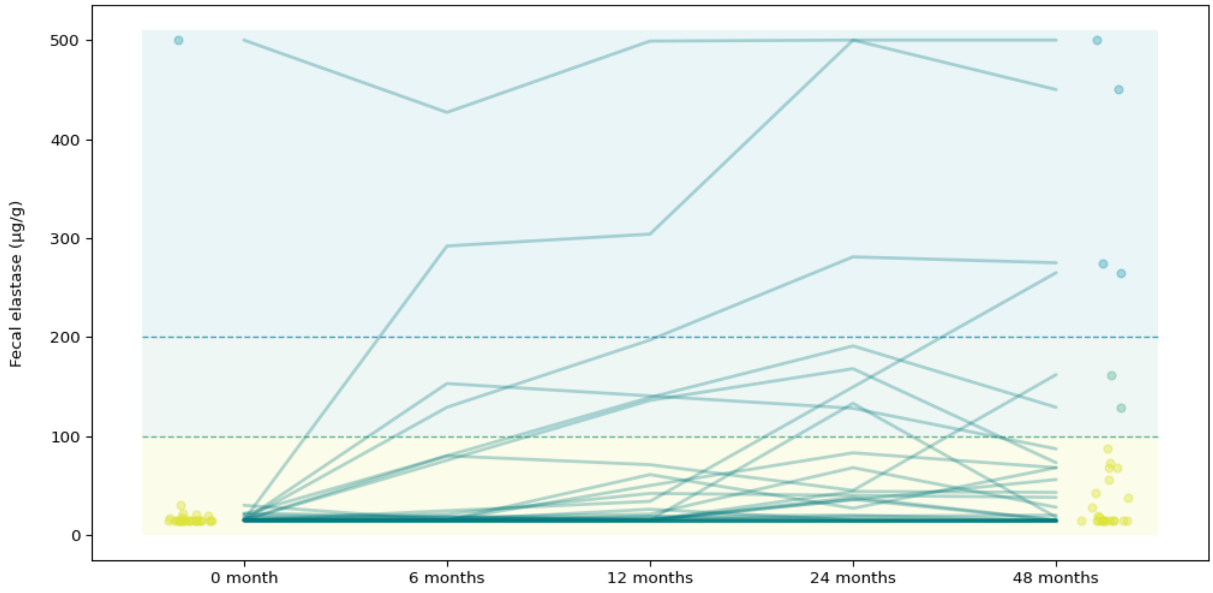

Suppl. Figure 4 *Pseudomonas aeruginosa* colonization in the year before the initiation of LUM/IVA therapy and in the second year of the follow-up

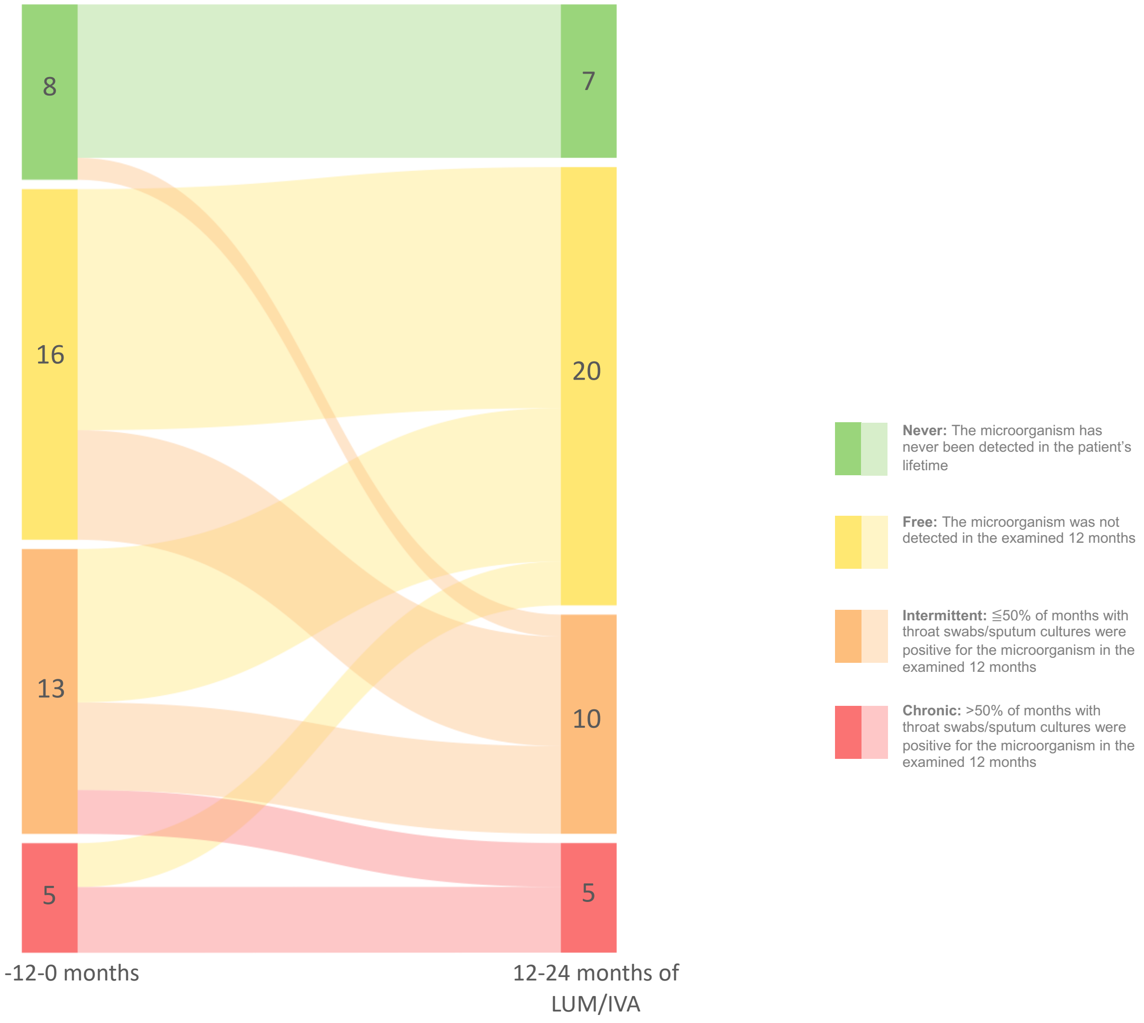

Supplement: Supplementary file 1 — Supplementary Information. [file 41598_2025_86010_MOESM1_ESM.pdf]
